# Blood collection in cell-stabilizing tubes does not impact germline DNA quality for pediatric patients

**DOI:** 10.1371/journal.pone.0188835

**Published:** 2017-12-05

**Authors:** Bruce M. Wollison, Edwin Thai, Aimee Mckinney, Abigail Ward, Andrea Clapp, Catherine Clinton, Anwesha Nag, Aaron R. Thorner, Julie M. Gastier-Foster, Brian D. Crompton

**Affiliations:** 1 Center for Cancer Genome Discovery, Dana-Farber Cancer Institute, Boston, Massachusetts, United States of America; 2 Institute for Genomic Medicine, Nationwide Children's Hospital, Columbus, Ohio, United States of America; 3 Dana-Farber/Boston Children's Cancer and Blood Disorders Center, Boston, Massachusetts, United States of America; 4 The Ohio State University College of Medicine, Columbus, Ohio, United States of America; 5 Cancer Program, Broad Institute of Harvard and MIT, Cambridge, Massachusetts, United States of America; The Ohio State University, UNITED STATES

## Abstract

**Objectives:**

Liquid biopsy technologies allow non-invasive tumor profiling for patients with solid tumor malignancies by sequencing circulating tumor DNA. These studies may be useful in risk-stratification, monitoring for relapse, and understanding tumor evolution. The quality of DNA obtained for these studies is improved when blood samples are collected in tubes that stabilizing white blood cells (WBC). However, ongoing germline research in pediatric oncology generally requires obtaining blood samples in EDTA tubes, which do not contain a WBC-stabilizing preservative. In this study, we explored whether blood samples collected in WBC-stabilizing tubes could be used for both liquid biopsy and germline studies simultaneously, minimizing blood collection volumes for pediatric patients.

**Methods:**

Blood was simultaneously collected from three patients in both EDTA and Streck Cell-Free DNA BCT^®^ tubes. Germline DNA was extracted from all blood samples and subjected to whole-exome sequencing and microarray profiling.

**Results:**

Quality control metrics of DNA quality, sequencing library preperation and whole-exome sequencing alignment were virtually identical regardless of the sample collection method. There was no discernable difference in patterns of variant calling for paired samples by either whole-exome sequencing or microarray analysis.

**Conclusion:**

Our study demonstrates that high-quality genomic studies may be performed from germline DNA obtained in Streck tubes. Therefore, these tubes may be used to simultaneously obtain samples for both liquid biopsy and germline studies in pediatric patients when the volume of blood available for research studies may be limited.

## Introduction

Strategies for applying liquid biopsy assays to the treatment of pediatric solid tumors is still in the early stages of development [1–4]. Studies are hampered by the limited number of patients available for enrollment in sample collection protocols at any one institution. Efforts to determine the prognostic value of liquid biopsy assays in pediatric solid tumors will, therefore, necessitate coordinated efforts across multiple institutions. These studies will require samples to be collected at institutions that may not be capable of rapidly processing samples locally and, instead, samples will need to be shipped and processed uniformly at a central laboratory. For this reason, many collaborative trials are beginning to utilize specialized WBC-stabilizing blood collection tubes for liquid biopsy studies. These tubes contain a nucleated cell-stabilizing preservative that prevent the lysis of WBCs when samples cannot be processed immediately. Stabilizing the WBCs prior to sample processing prevents the release of large amounts of genomic DNA into the plasma that would otherwise dilute circulating tumor DNA (ctDNA) levels to undetectable proportions.

In pediatrics, the volume of blood drawn for research is sometimes restricted to ensure the safety of young children who have smaller blood volumes and may be more hemodynamically affected by sample collections. Thus, researchers must often choose between collecting samples for one research project over another. We anticipate that this may limit the usefulness of requesting that additional blood be collected in WBC-stabilizing tubes in ongoing research efforts in pediatric oncology. Therefore, we sought to determine whether samples obtained in WBC-stabilizing tubes could be used simultaneously for two purposes: 1) liquid biopsy studies and 2) germline studies. While the use of WBC-stabilizing tubes for ctDNA studies have been well-validated, to our knowledge there are no published studies describing the quality of germline DNA obtained from these tubes and their usefulness in performing germline genomic analyses. Here, we describe our experience performing whole-exome sequencing (WES) and genotyping by copy number and single nucleotide polymorphism (SNP) array analysis of germline samples obtained simultaneously in standard EDTA and Streck Cell-Free DNA BCT^®^ collection tubes from three patients.

## Methods

### Patients and consent

Blood samples were collected from patients enrolled on a Dana-Farber Cancer Institute IRB-approved banking study (DFCI protocol #11–104). Written informed consent was obtained from participants or parents of minor participants prior to study inclusion. Patients were selected for participation based on an expectation that they would have normal or near-normal WBC counts based on their clinical course. We also chose children older than 11 years old for the study to ensure that drawing multiple tubes of blood simultaneously would not infringe on the hospital established blood volume limits as it would in younger patients. We chose patients who were expected to require venous access for clinical purposes so that no additional procedures were required to obtain these samples. For each patient, 5 mL of blood was drawn into a purple top EDTA tube immediately followed by the collection of 10 mL of blood into a Streck Cell-Free DNA BCT^®^ tube.

### Sample processing and DNA extraction

Blood samples were processed at the Boston Children’s Hospital Biorepository on the same day as the specimens were acquired. For blood collected in both EDTA and Streck tubes, samples were first centrifuged at 1000 x g at 4° Celsius for 10 minutes in the collection tube. Plasma was then removed and stored for future use. The remaining blood, a combination of red blood cells and white blood cells, were then removed from the collection tube and subjected to DNA extraction using the Gentra Puregene Blood Kit (Qiagen) according to the manufacturer’s instructions. DNA was quantified for each sample using the Quant-iT PicoGreen dsDNA Assay Kit (Thermo Fisher Scientific).

### Whole exome library construction and sequencing

Control DNA for this experiment was obtained from the human B-lymphocyte cell line CEPH1408 (GM10831), (Catalog No. NA10831; Coriell Institute for Medical Research) [5]. A total of 100 ng of DNA from each sample was ultra-sonicated to an average fragment size of 250 base pairs (Covaris) and further size selected using Agencourt AMPure XP beads (Beckman Coulter). DNA was then manually ligated to adaptors using the KAPA HTP Library Preparation Kit (KK8234, KAPA Biosystems). All libraries were purified and size selected using Agencourt AMPure XP beads to perform a double-sided solid phase reversible immobilization (SPRI) cleanup. The bead to DNA ratio of 0.5X was used for the high molecular weight SPRI selection followed by a 2.5X SPRI selection. Libraries were analyzed using a Bioanalyzer (Agilent) to ensure that all sample libraries had the expected DNA fragment size distribution. Library concentrations were quantitated using an Illumina MiSeq. Briefly, 1 μl from each library was pooled and quantitated using a Kapa Biosystems Library Quant Kit (KK4854, Kapa Biosystems), normalized to 4 nM, and then sequenced on a MiSeq nano flow cell (Illumina). The “sequenceable” concentration of each library was calculated by dividing the number of clusters per barcode by the average number of clusters and then multiplying the result by the qPCR value of the pooled samples. The libraries were then normalized and pooled for hybrid capture such that samples were divided into two capture reactions performed with a total of 750 ng of pooled library DNA. Capture was performed using the SureSelectXT Hybrid Capture kit with the Exon v5 bait set (Agilent Technologies). After capture, all samples were pooled together and sequenced across two lanes of a Hiseq 2500 in rapid run mode (Illumina).

### Sequencing analysis

Pooled sample reads were deconvoluted and sorted using Picard tools (http://broadinstitute.github.io/picard/picard-metric-definitions.html). Reads were then aligned to the b37 edition of the human genome reference sequence (Human Genome Reference Consortium) using bwa (http://bio-bwa.sourceforge.net/bwa.shtml) utilizing the parameters “-q 5 -l 32 -k 2 -o 1”. Duplicate reads were identified and removed using Picard tools [6]. Alignments were further refined using GATK for localized realignment around sites of small insertions and deletions (https://www.broadinstitute.org/gatk/gatkdocs/org_broadinstitute_gatk_tools_walkers_indels_IndelRealigner.php). Base quality score recalibration was also performed using GATK (http://gatkforums.broadinstitute.org/discussion/44/base-quality-score-recalibration-bqsr) [7, 8]. Unaligned reads were used to confirm performance of both sequencing lanes. We then determined the number of reads per sample, mean target coverage, and percent of targeted bases with at least 30x sequencing coverage to estimate the sequencing quality across all samples. Lastly, DNA fingerprinting analysis was performed using Picard Tools to confirm concordance at 44 polymorphic loci among samples obtained from the same patient.

Variant analysis for single nucleotide variants (SNV) was performed using MuTect v1.1.4 and annotated by Variant Effect Predictor (VEP). The SomaticIndelDetector tool from GATK was used for indel calling [9, 10]. MuTect was run in paired mode using sequencing data generated from human cell line CEPH1408 as the project normal. Samples were compared to identify variant calls that were discordant between samples collected in EDTA and Streck tubes from the same patient and to investigate whether any patterns of sequencing discrepancies could be appreciated between the two different collection methods. Aligned BAM files are available at the NCBI Sequencing Read Archive at https://trace.ncbi.nlm.nih.gov/Traces/sra/?study=SRP120033.

### Copy number microarray

Microarray analysis was performed on 1 ug of DNA sample. Each test sample was digested in parallel with a sex matched control (Agilent Male/Female Reference DNA) using the restriction enzymes Alu I and RSA I to obtain 200-500bp fragments. Following digestion, the test samples were labeled with Cyanine 5-dUTP and control samples were labeled with Cyanine 3-dUTP. The unincorporated dye was removed. Each test sample and correlating control were combined and hybridized to a custom 4x180K CGH plus SNP microarray (GGXChip+SNP v1.0, Agilent). The copy-number probes on the array (~135,000) have an average spacing of one probe every 35 kb throughout the genome and one probe every 10 kb in regions known to have clinical significance while the SNP probes (~67,000) have an average spacing of 40 kb across the genome. Slides were scanned on a SureScan Microarray Scanner (Agilent) and data was analyzed through CytoGenomics v4.0.3.1.2 (Agilent) and Genoglyphix v3.1 (Perkin Elmer). Array data can be accessed at the NCBI Gene Expression Omnibus site https://www.ncbi.nlm.nih.gov/geo/query/acc.cgi?acc=GSE105032.

## Results

### Genomic DNA can be extracted from blood samples collected in Streck tubes

Genomic DNA was obtained from blood collected in both EDTA and Streck blood collection tubes using a standard extraction kit with no modifications to the protocol for either tube. In all samples, we extracted over 100 μg of genomic DNA material using these standard procedures (Table 1). In most cases, the Streck tube yielded a larger amount of genomic DNA per sample, likely due to the larger starting blood volume compared to samples collected in the EDTA tube. Aliquots of 100 ng of DNA were then used for DNA sequencing and 1 μg was used for copy number analysis. Over 100 μg of genomic DNA material from each sample remained available for additional studies, demonstrating that blood collected in both EDTA and Streck tubes yield ample DNA for multiple genomic studies.

**Table 1:** DNA extraction and sequencing metrics.

Subject ID	Sample Type	DNA extracted (μg)	DNA (ug) per mL blood	Library Yield (ng)	Reads	Mean Target Cvg (x)	% Target Bases 30x
179	EDTA	230.1	46.0	2242.19	71,417,612	71.7	89.2
	Streck	399.0	39.9	3060.21	84,343,168	83.2	92
394	EDTA	349.3	69.9	2615.99	90,165,770	87	93.2
	Streck	513.3	51.3	2909.82	90,655,396	87.6	93.2
403	EDTA	158.2	31.6	2985.58	91,215,682	87.2	93.2
	Streck	144.1	14.4	3313.12	83,573,048	81.5	91.7
CEPH1408	Cell line			3440.64	81,051,808	80.5	91.8

DNA extraction and sequencing metrics for whole-exome sequencing of germline DNA extracted from blood samples collected in EDTA and Streck tubes.

### High-quality sequencing can be performed from blood samples collected in Streck tubes

Sequencing libraries were prepared from 100 ng of genomic DNA from each sample. All sequencing libraries were prepared and purified uniformly but with unique barcodes so that samples could be multiplexed for sequencing and then de-multiplexed during analysis. Library yields from all blood samples were remarkably similar and were comparable with the DNA library yields generated from DNA extracted from a human cell line, CEPH1408 (Table 1). Bioanalyzer electropherograms demonstrated that all samples had uniform and expected sample library fragment size (data not shown).

Recently published cancer predisposition studies have used both whole-genome sequencing and targeted sequencing approaches to identify recurrent germline variants in patients and families with a high incidence of cancer [11–15]. Targeted sequencing often involves a hybrid capture step to enrich sequencing libraries for genomic regions of interest. Thus, hybrid capture of DNA libraries was performed on all samples to enrich for the coding region of the genome using the Agilent Exon v5 capture bait set. Captured libraries were then sequenced in order to achieve an expected target coverage of approximately 80x.

**Fig 1:**
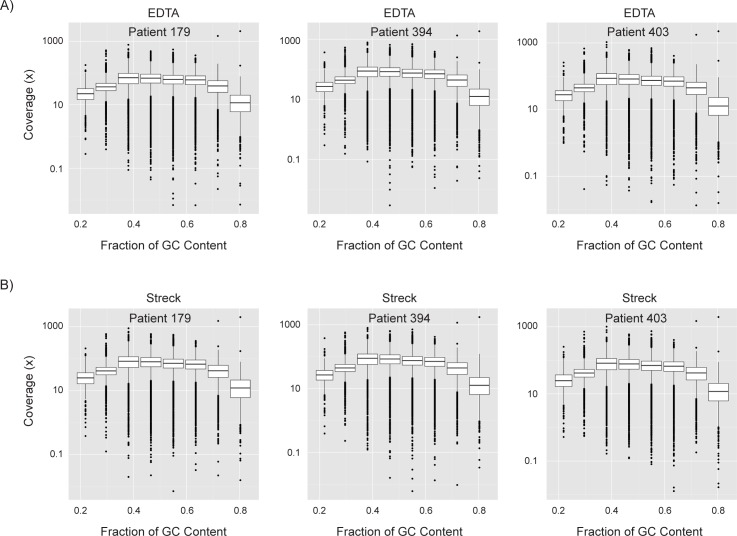
Sequencing coverage is not effected by sample collection tube. **(A-B)** Box plots of the read coverage for target regions binned by GC content for samples collected in **A)** EDTA tubes or **B)** Streck tubes. For all box plots, the central box indicates values in the range 25^th^-75^th^ percentile of all values for that subset of data with the central line indicating the median. Whiskers extend 1.5x from the lower and upper boundaries of the central box with points outside that range indicated as black circles.

There were a similar number of total sequencing reads generated per sample (Table 1). Plasma samples had an average sequencing depth of 83x (71.7x – 87.6 x) for the target regions, and over 89% of all targeted bases had at least 30x coverage (Table 1). WES metrics were similar regardless of whether genomic DNA samples had originated from blood collected in an EDTA or Streck tubes. When read coverage was binned by GC content of the targeted regions, mean coverage was nearly identical between paired samples obtained in EDTA tubes and samples collected in Streck tubes, and differences in normalized mean coverage were less than 0.02x for all categories of percent GC content (Fig 1).

**Fig 2:**
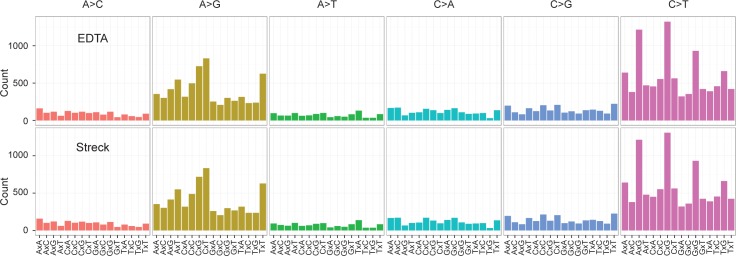
Mutation pattern is not effected by sample collection. **(A-B)** Lego plot of single-nucleotide variants plotted by sequence context for variants with allelic fractions > 0.05 and < 0.95. Samples collected in **A)** EDTA tubes and **B)** Streck tubes were combined and plots show the sum of the three samples.

Sequence variants were identified by comparing each sample to DNA extracted from a human cell line, CEPH1408, which was sequenced simultaneously with the germline samples. A similar number of unfiltered variants were identified for each patient, regardless of whether the germline sample sequenced was collected in an EDTA or Streck tube (Table 2). Since all germline variants can be expected to be either heterozygous or homozygous, we expected true variants to have allelic fractions close to 0.5. However, because hybrid capture sequencing may introduce some sequencing bias between alleles, we included in our analysis all variants with an allelic fraction > 0.25 and sequencing coverage of greater than 30 reads [16, 17]. All samples had a similar percentage of variants that passed this filter regardless of which tube the sample was used for blood collection (57.1–67.1%; Table 2). When each sample was compared to the matched sample from the same patient, less than 1.2% of the variants were unique to the individual sample, consistent with the published rates of sequencing error observed when repeated WES has been performed [18–21]. Furthermore, when variants were filtered by whether they were seen in any of the other 5 samples sequenced, less than 0.58% of variants were unique to that sample, demonstrating an extremely low sequencing error rate that was similar regardless of the type of tube in which the sample was collected. Finally, we compared the pattern of mutations observed in each sample across the three base-pair context and could not detect a bias in mutation pattern between samples collected in EDTA or Streck tubes (Fig 2).

**Table 2:** WES variant calls.

Subject ID	Sample Type	Total Variants	Filtered (% of Total Variants)	Filtered Not in Pair (% Filtered)	Filtered Unique (% Filtered)
179	EDTA	21672	12379 (57.12%)	122 (0.99%)	59 (0.48%)
	Streck	22598	14579 (64.51%)	175 (1.2%)	84 (0.58%)
394	EDTA	25478	17153 (67.32%)	182 (1.06%)	89 (0.52%)
	Streck	25147	16452 (65.42%)	152 (0.92%)	76 (0.46%)
403	EDTA	25213	16909 (67.06%)	176 (1.04%)	85 (0.5%)
	Streck	24620	15587 (63.31%)	146 (0.94%)	65 (0.42%)

Summary of sample variants called from whole-exome sequencing of germline DNA compared to a cell line control. Total variants were subsequently filtered using three increasingly stringent criteria: 1) Filtered variants were retained if the allelic fraction was > 0.25 and the coverage was > 30x 2) Filtered Not in Pair variants were retained if the variant was not seen in the other patient-matched sample 3) Filtered Unique variants were retained if the variant was not seen in any of the other samples.

### Genome-wide copy number plus SNP microarrays identify the same variants in germline samples regardless of sample collection strategy

DNA samples were also analyzed with genome-wide combined copy number/SNP mircroarrays. For each patient, regions of copy number alteration and homozygosity were identified and compared between samples obtained in EDTA tubes and Streck tubes. The quality control metrics were excellent for copy number analysis using the derivative log ratio spread (DLR) metric and SNP analysis using the call rate (CR) metric, regardless of how the sample was collected (Table 3). Copy number analysis showed that clinically relevant data was identical for the Streck and EDTA-derived samples. Similarly, matched samples showed identical regions of homozygosity (Table 3).

**Table 3:** Microarray results.

Subject ID	Sample Type	DLR	SNP CR	% Overlapping CN Calls	SNP Calls
179	EDTA	0.1261	0.95	8p11.22 loss, 14q32.33 gain,16p12.2 gain, Xp22.33 loss	none
	Streck	0.1249	0.937	8p11.22 loss, 14q32.33 gain,16p12.2 gain, Xp22.33 loss	none
394	EDTA	0.1241	0.971	6p25.3 loss, 7q11.21 loss, 8p11.22 gain,22q11.22 gain	none
	Streck	0.1295	0.972	6p25.3 loss, 7q11.21 loss, 8p11.22 gain,22q11.22 gain	none
403	EDTA	0.1292	0.977	1q44 loss, 7p22.3 gain, 8p11.22 loss, 14q32.33 gains, 15q11.2,17q21.31 loss	2p25.1–24.3 ROH, 3p24.3-p24.1 ROH, 6p21.2-p12.1 ROH,7q31.1-q31.31 ROH
	Streck	0.1322	0.946	1q44 loss, 7p22.3 gain, 8p11.22 loss, 14q32.33 gains, 15q11.2,17q21.31 loss	2p25.1–24.3 ROH, 3p24.3-p24.1 ROH, 6p21.2-p12.1 ROH,7q31.1-q31.31 ROH

Germline DNA samples were tested on a custom combined copy number plus single nucleotide polymorphism microarray. Sample-specific quality metrics for copy number (DLR) and SNP (SNP call rate) are shown along with copy-number calls and SNP calls (ROH > 5 Mb).

## Discussion

The use of liquid biopsy assays that detect, quantify, and profile ctDNA are being routinely incorporated into clinical trials for adult cancers [22–24]. These assays can be used to detect targetable mutations in a patient’s cancer, sometimes without the need for invasive biopsies [25, 26]. Changes in ctDNA levels often correspond to changes in disease burden and thus, these assays have also been used to track response to therapy and to monitor for relapse [27–29]. Profiling of ctDNA has been used to detect the emergence of resistance mutations in tumors for patients on targeted therapy and efforts are now underway to perform more expansive profiling of ctDNA samples to define patterns of clonal evolution and treatment resistance [24, 25, 30].

Such efforts in pediatric cancers are also beginning to emerge [1–4]. Published studies of ctDNA in pediatric solid tumors have utilized relatively small cohorts of patients, justifying further study of ctDNA. However, these initial efforts have been insufficiently powered to study the prognostic value of these assays. Studies in adult cancers confirm that ctDNA levels vary widely between cancer types and assay designs are also dependent on the expected genomic landscape of each histology [22]. Thus, studies in pediatric solid tumors will require focused, disease-specific efforts. Due to the relative rarity of most pediatric solid tumors, the development and validation of liquid biopsy technologies will require collaborative efforts that extend beyond single institutions. There are several existing collaborative studies underway that would be uniquely poised to adequately collect blood samples and clinical data, including but not limited to, the Children’s Oncology Group (COG) Project: Every Child (https://clinicaltrials.gov/ct2/show/NCT02402244), the GAIN Consortium’s iCAT2 study (https://clinicaltrials.gov/ct2/show/NCT02520713), and the NCI-MATCH study for pediatrics (https://www.cancer.gov/about-cancer/treatment/clinical-trials/nci-supported/pediatric-match).

There are several logistical hurdles to overcome in developing liquid biopsy technologies in trials enrolling pediatric patients at multiple institutions. One major issue is that samples must be collected and shipped to a central laboratory before being processed. ctDNA is easily extracted from plasma isolated from blood collected in EDTA tubes using standard extraction kits. However, several studies have demonstrated that plasma must be isolated from blood collected in EDTA tubes within eight hours of collection to prevent the chance of lysis of WBCs that will contaminate the plasma with genomic DNA [31–35]. This genomic DNA impairs the ability to easily detect and quantify ctDNA from the plasma by lowering the ctDNA abundance relative to germline DNA in the sample. One way to circumvent this problem is to collect blood in preservative-containing tubes designed to stabilize WBCs, thereby allowing samples to be collected at numerous institutions and then be shipped to a central laboratory for processing. Furthermore, liquid biopsy studies have demonstrated that ctDNA can be more easily detected from larger volumes of plasma. Thus, clinical trials designed to study ctDNA in collaborative efforts would benefit from collecting large volumes of blood. However, another logistical hurdle in pediatrics is that many of our patients have much smaller blood volumes, restricting the amount of blood that can be collected at any one-time point.

One competing research priority in pediatrics is the collection of a blood sample for germline DNA profiling. DNA for these studies are most often extracted from WBCs obtained from blood collected in an EDTA tube. A potential solution for collecting plasma for ctDNA studies while still prioritizing the collection of germline material for genetic studies would be to utilize a single tube of blood for both germline and liquid biopsy studies. To determine the feasibility of this approach, we collected blood from three patients simultaneously in EDTA and Streck tubes and performed WES and SNP-arrays on the matched germline DNA extracted from cells from each tube. We demonstrated that the WES and SNP-array data was of very similar quality for germline studies, suggesting that both ctDNA and germline study samples can be collected from a single WBC-stabilizing tube for pediatric cancer patients. Similar results were observed when utilizing column-based extraction methods to obtain genomic DNA from EDTA and Streck tubes (data not shown). One potential limitation of this study is that we were not able to test the effects of exposing blood samples to a wider range of temperatures or processing times. However, guidelines for handling Streck tubes suggest that samples be maintained at room temperature during shipment and processed within 7–10 days. We would expect that conforming to those guidelines would ensure the quality of cell-free and germline DNA extracted from blood collected in Streck tubes. We believe this data will facilitate the collection of liquid biopsy studies on multi-institutional trials without compromising germline studies or increasing the blood volumes requested for ongoing research.
